# Small intestinal flukes of the genus *Metagonimus* (Digenea: Heterophyidae) in Europe and the Middle East: A review of parasites with zoonotic potential[Fn FN1]

**DOI:** 10.1051/parasite/2024016

**Published:** 2024-03-29

**Authors:** Tomáš Scholz, Roman Kuchta, Daniel Barčák, Gábor Cech, Mikuláš Oros

**Affiliations:** 1 Institute of Parasitology, Biology Centre, Czech Academy of Sciences Branišovská 31 370 05 České Budějovice Czech Republic; 2 Institute of Parasitology, Slovak Academy of Sciences Hlinkova 3 040 01 Košice Slovakia; 3 HUN-REN Veterinary Medical Research Institute Hungária krt. 21 1143 Budapest Hungary

**Keywords:** Trematoda, Taxonomy, Redescription, SEM, Genotyping, Fish-borne zoonoses, Palaearctic region

## Abstract

The heterophyid trematode *Metagonimus romanicus* (Ciurea, 1915) (Digenea) is redescribed on the basis of type material from domestic dogs (*Canis familiaris*) in Romania, vouchers from experimentally infected cats (*Felis catus*) and adults recovered from golden hamsters (*Mesocricetus auratus*) infected with metacercariae from scales of chub (*Squalius cephalus*) and common nase (*Chondrostoma nasus*) (Cypriniformes: Leuciscidae) in Hungary. This trematode, endemic to Europe and neighbouring regions (northwestern Türkiye), was previously misidentified as *M. yokogawai* (Katsurada, 1912), a zoonotic parasite of humans in East Asia. However, the two species differ considerably both genetically and morphologically, *e.g*., in the position of the ventral sucker, the presence of the prepharynx, the anterior extent of the vitelline follicles and the posterior extent of the uterus. *Metagonimus ciureanus* (Witenberg, 1929) (syn. *Dexiogonimus ciureanus* Witenberg, 1929), described from domestic cats and dogs in Israel, is a valid species distributed in the Middle East and Transcaucasia, which is also confirmed by molecular data. It differs from all *Metagonimus* species, including *M. romanicus*, in having symmetrical testes instead of the oblique testes of the other congeners. The zoonotic significance of *M. romanicus* and *M. ciureanus* is unclear, but appears to be low in Europe, mainly because raw or undercooked, whole fish with scales are generally not consumed. Accidental infection of fishermen by metacercariae in the scales when cleaning fish is more likely, but has never been reported. Remains of cyprinoids with scales infected with metacercariae of *Metagonimus* spp. can be an important natural source of infection for dogs, cats, and other carnivores, which can serve as a reservoir for these parasites.

## Abstract (fr)

Le trématode Heterophyidae *Metagonimus romanicus* (Ciurea, 1915) (Digenea) est redécrit sur la base de matériel type provenant de chiens domestiques (*Canis familiaris*) en Roumanie, de vouchers issus de chats (*Felis catus*) infectés expérimentalement et d’adultes collectés chez des hamsters dorés (*Mesocricetus auratus*) infectés par des métacercaires provenant d’écailles de chevesne commun (*Squalius cephalus*) et de nase commun (*Chondrostoma nasus*) (Cypriniformes : Leuciscidae) de Hongrie. Ce trématode, endémique d’Europe et des régions voisines (nord-ouest de la Turquie), avait été précédemment identifié à tort comme étant *M. yokogawai* (Katsurada, 1912), un parasite zoonotique des humains en Asie de l’Est. Cependant, les deux espèces diffèrent considérablement sur le plan génétique et morphologique, par exemple par la position de la ventouse ventrale, la présence du prépharynx, l’étendue antérieure des follicules vitellins et l’étendue postérieure de l’utérus. *Metagonimus ciureanus* (Witenberg, 1929) (syn. *Dexiogonimus ciureanus* Witenberg, 1929), décrite chez des chats et des chiens domestiques en Israël, est une espèce valide répartie au Moyen-Orient et en Transcaucasie, ce qui est également confirmé par des données moléculaires. Cette espèce diffère de toutes les espèces de *Metagonimus*, y compris *M. romanicus*, par ses testicules symétriques au lieu des testicules obliques des autres congénères. L’importance zoonotique de *M. romanicus* et *M. ciureanus* n’est pas claire, mais semble faible en Europe, principalement parce que les poissons n’y sont généralement pas consommés crus ou insuffisamment cuits et entiers avec les écailles. L’infection accidentelle des pêcheurs par des métacercaires présents dans les écailles lors du nettoyage du poisson est plus probable mais n’a jamais été signalée. Les restes de poissons cyprinoïdes avec les écailles, infectés par des métacercaires de *Metagonimus* spp. peuvent être une source naturelle importante d’infection pour les chiens, les chats et autres carnivores, qui peuvent servir de réservoir à ces parasites.

## Introduction

Fish-borne parasitic diseases are a public health problem in regions where raw or undercooked fish is consumed. Most human cases are caused by two groups of trematodes (Digenea): (i) small liver flukes from the family Opisthorchiidae [74, 83], including *Opisthorchis viverrini* (Poirier, 1886), which can cause cholangiocarcinoma in Southeast Asia [9]; and (ii) small intestinal flukes, most of which belong to the family Heterophyidae, with the most common causative agents of human disease belonging to the genera *Haplorchis* Looss, 1899; *Heterophyes* Cobbold, 1886; *Stellantchasmus* Onji & Nishio, 1916; and *Metagonimus* Katsurada, 1912 [33, 35].

Seven species of the latter genus can infect humans, almost exclusively in East Asia, with *M. yokogawai* (Katsurada, 1912), *M. takahashii* Suzuki in Takahashi, 1929, *M. miyatai* Saito, Chai, Kim, Lee et Rim, 1997, and *M. suifunensis* Shumenko, Tatonova et Besprozvannykh, 2017, which are epidemiologically most important because they have a high prevalence and intensity of infection in people living in endemic areas [34, 67, 81, 88]. However, *M. yokogawai* has also been reported from Europe (mainly from fish in the countries around the Danube, Dniester and Dnieper rivers) and from mammals and birds in the Middle East and Transcaucasia [10, 19, 39, 42, 46, 48, 65, 84].

However, Cech *et al*. (2023) [12] provided molecular evidence that *Metagonimus* trematodes in fish from the Hungarian Danube were misidentified as *M. yokogawai*, which most likely does not occur in Europe. These authors presented DNA sequences of metacercariae from different cyprinoid species as well as adults from experimentally infected hamsters and chicks, and showed that they differ from those of all Asian *Metagonimus* species, including *M. yokogawai*. Cech *et al*. [12] also suggested that these trematodes, previously misidentified as *M. yokogawai*, might belong to the little-known *M. romanicus* (Ciurea, 1915) from Romania, but provided no morphological evidence. According to Chai and Jung (2024) [34], the taxonomic position of *M. romanicus* needs further investigation.

Recently, new material of metacercariae was collected in the Hungarian part of the Danube and adults were obtained from experimentally infected hamsters and chicks. In addition, the type specimens (syntypes) and voucher specimens of *M. romanicus* (Ciurea, 1915) were examined. This enabled us to confirm the species identity of the trematodes from the Hungarian Danube and to redescribe the little-known European species *M. romanicus* in the present study.

Another species, *M. ciureanus* (Witenberg, 1929), was described from the Middle East (Israel) and later reported from Transcaucasia [79, 94]. In this study, we examined type and voucher specimens of *M. ciureanus* and provided redescription of this little-known species with a limited distribution range, but with zoonotic potential due to its occurrence in commercially important fish such as tilapias [94].

## Materials and methods

### Morphological study

The following specimens of *Metagonimus romanicus* were examined: (1) 20 syntypes, including five adult specimens that were stained and whole-mounted, and two that were used for scanning electron microscopy (SEM) examination, from the domestic dog *Canis familiaris* L., Somova, Tulcea County, Romania, collected by J. Ciurea, July 1914 (National Museum of Natural History, Smithsonian Institution, Washington, D.C., USA – USNM 1327535); (2) 10 specimens, including five newly stained and whole mounted specimens, from the domestic dog, Bucharest, Romania, J. Ciurea, December 23, 1913 (USNM 1341217); (3) 17 adult specimens (8 days post-infection – DPI) from golden hamsters *Mesocricetus aureus* Waterhouse, fed with metacercariae from scales of chub *Squalius cephalus* (L.) (Helminthological Collection of the Institute of Parasitology, Biology Centre of the Czech Academy of Sciences, České Budějovice, Czech Republic – IPCAS D-876/1); (4) adults (8 DPI) from a golden hamster fed with metacercariae from scales of common nase *Chondrostoma nasus* (L.) (both Cypriniformes: Leuciscidae) (IPCAS D-876/1); (5) three adults (10 DPI) from chicks *Gallus domesticus* L., fed with metacercariae from scales of chub (IPCAS D-876/2), all fish from the Danube in Szentendre, north of Budapest, Hungary (47.663969N, 19.079874E), collected on April 24 and 25, 2023.

The following specimens, identified as *Metagonimus yokogawai*, were also examined: (1) five specimens from purple heron *Ardea purpurea* L., night heron *Nycticorax nycticorax* (L.) and great cormorant *Phalacrocorax carbo* (L.), all from Krasna Hatka, Cherson Oblast, Ukraine, collected by L.A. Smogorzhevskaya on July 2 and 1, and June 22 1952, respectively (Schmalhausen Institute of Zoology, Kyiv, Ukraine – Coll. Nos. 879, 880 and 931; see [86]); (2) 35 specimens from domestic cats *Felis catus* L., experimentally infected with metacercariae from scales of cyprinoids in Kamenica nad Hronom, Slovakia, collected by R. Žitňan on November 30, 1958 (East Slovak Museum, Košice, Slovakia – ESM Z-11235); (3) four adult *M. yokogawai* from white-tailed eagle *Haliaeetus albicilla* (L.), from Třeboň, South Bohemia, Czech Republic, J. Sitko, April 18, 2004 (IPCAS D-422/2; Comenius Museum Přerov, Czech Republic – CMP 10.883-5); (4) 8 specimens from a golden hamster (16 DPI) fed with metacercariae from muscles of ayu sweetfish *Plecoglossus altivelis* (Temminck and Schlegel), Ai River near Urakawa, Sakuma, Shizuoka Prefecture, Honshu, Japan, H. Kino, August 2004 (IPCAS D-422/1); (5) one adult specimen from a great cormorant, *Phalacrocorax carbo*, Zalew Wiślany, Poland, May 9, 2005, G. Kanarek; (6) four adults from a golden hamster (20 DPI) fed with metacercariae from scales of barbel steed *Hemibarbus labeo* (Pallas), Lake Biwa, Shiga Prefecture, Japan, T. Shimazu, June 20, 2006 (IPCAS D-422/1); and (7) 30 adult *M. yokogawai* from domestic dogs (37 DPI) fed with metacercariae from scales of *H. labeo*, Lake Biwa, Shiga Prefecture, Japan, T. Shimazu, July 16, 1980 (IPCAS D-422/1).

The following specimens of *Metagonimus ciureanus* (syn. *Dexiogonimus ciureanus*) were examined: (1) 57 syntypes, including nine stained and whole-mounted specimens and three used for SEM examination by the present authors, from the domestic cat, *F. catus*, Jaffa, Israel, G. Witenberg, 1928 (Natural History Museum, London, United Kingdom – NHMUK 1929.7.24.55–74, 2000.4.10.103); (2) one specimen (voucher) from *F. catus*, Jordan, S. Abdel-Hafez (NHMUK 1983.1.11.2) [40]; (3) 12 specimens from the domestic cat (*F. catus*) identified as *M. ciureanus* in Sapanca, Türkiye, R. E. Kuntz, summer 1953 (USNM 1339602) [16].

The following Asian species of *Metagonimus* were also examined: nine paratypes of *M. hakubaensis* Shimazu, 1999 from experimentally infected brown rat, *Rattus norvegicus* (Berkenhout) (22 DPI), T. Shimazu, July 16, 1996 (IPCAS D-416/1); four adult *M. hakubaensis* from the golden hamster (14 DPI) fed with metacercariae from the Far Eastern brook lamprey, *Lethenteron reissneri* (Dybowski), Hime River near Sano, Hakuba, Japan, T. Shimazu (IPCAS D-416/2); four adult *M. miyatai* Saito, Chai, Kim, Lee et Rim, 1997 from golden hamster (14 DPI) fed with metacercariae from *Phoxinus steindachneri* Sauvage, Kotobuki, Iiyama, Nagano Prefecture, Japan, T. Shimazu, August 21, 2004 (IPCAS D-555/1); three adult *M. otsurui* Saito et Shimizu, 1968 from golden hamster (14 DPI) fed with metacercariae from *Rhinogobius flumineus* (Mizuno), Takami River at Kotsukawa, Higashiyoshino, Nara Prefecture, Japan, T. Shimazu, August 7 and 8, 2004 (IPCAS D-554/1); and four adult *M. takahashi* Suzuki, 1930 from golden hamster (14 DPI) fed with metacercariae from goldfish, *Carassius auratus* (L.), Lake Suwa near Suwa, Nagano Prefecture, Japan, T. Shimazu, June 12, 2004 (IPCAS D-553/1).

The experiments with metacercariae to obtain adult parasites were performed as described in detail by Cech *et al*. (2023) [12]. In brief, the metacercariae were isolated from the fish scales, morphologically identified (they corresponded in morphology to those molecularly characterised by Cech *et al*. [12]) and used for experimental infections. Just one type of metacercariae was found in the fish scales. The 28S sequences of four randomly selected metacercariae from the chub, *Squalius cephalus* (L.), were identical to those of metacercariae and adults studied by Cech *et al*. [12] and adults obtained by the present authors.

Six golden hamsters and three one-day-old chicks were infected with the metacercariae from *S. cephalus* and common nase, *Chondrostoma nasus* (L.) (Cyprinoidei: Leuciscidae), and necropsied as described by Cech *et al.* (2023) [12] (permit number 070/PP/SOVII/2023; CZ04094). Live adults found in the gut were carefully washed in saline and, depending on their numbers, fixed in hot (almost boiling) 4% formaldehyde solution (formalin) or in hot saline and then placed in “cold” (= laboratory temperature) formalin, or in 70% molecular ethanol or directly in 96% molecular ethanol for DNA sequencing.

Selected adult worms were stained with Mayer’s carmine, dehydrated in an ethanol series, clarified with eugenol (clove oil) and embedded as permanent preparations in Canada balsam. The dimensions in the morphological descriptions are given in micrometres. The drawings were made with a drawing attachment on an Olympus BX51 microscope with Nomarski interference contrast.

Several specimens of *M. romanicus* and *M. ciureanus* were prepared for SEM following the procedure of Hernández-Orts *et al*. [29].

### Molecular study

The specimens selected for the phylogenetic study were isolated from two golden hamsters experimentally infected in April 2023 with encysted metacercariae from the scales of *Squalius cephalus* and *Chondrostoma nasus*, respectively, both from the Danube near Szentendre, Hungary. Total genomic DNA was extracted from six adult specimens with eggs using an innuPREP DNA kit (Analytik Jena, Jena, Germany) according to the manufacturer’s instructions, eluting with 35 μL of ultrapure water.

Three genetic markers, the nearly complete small subunit of ribosomal RNA gene (18S), the D1–D3 region of large subunit of ribosomal RNA gene (28S) and the complete gene of mitochondrial cytochrome c oxidase subunit I (COI), were amplified using TaKaRa Ex Taq HS (TaKaRa, Shiga, Japan). With the exception of annealing temperatures, PCR conditions were identical for all markers: initial denaturation at 95 °C for 3 min, 35 cycles of denaturation at 95 °C for 30 s, annealing at 55 °C (18S) or at 56 °C (28S, COI) for 30 s, elongation at 72 °C for 1 min, and final elongation at 72 °C for 7 min. The primers Worm A and Worm B [54] were used for amplification of the 18S, the primers ZX1 and 1500R [5, 69] for the 28S, and the modified primers MPF26 (5′-CTGTCTTCAAAACGGGAGG-3′) [87] and MetCOI_R4 (5′-CATGATGCAAAAGGTACAASAC-3′) [63] for amplification of the complete COI gene.

The PCR products were verified by electrophoresis on 1% agarose gel and enzymatically purified [93]. Sanger dideoxy sequencing was performed by SEQme (Dobříš, Czech Republic) using PCR and the internal primers 600R [54] and 1600F (5′-CCAGGTCTGTGATGCCC-′3) for 18S, 400R and 900F [69] for 28S, and MetCOI 600R (5′-GCGATCAAAAAGAAGCATCG-3′) and MetCOI 600F (5′-CGATGCTTCTTTTTGATCGC-3′) for COI. New sequences were generated by *de novo* assembly of four Sanger reads; primer-complementary parts were cut-off from ribosomal gene sequences, and start (GTG) and stop (TAG) codons were recognised to delineate the COI gene. Thirteen newly generated sequences with a length of 1,830 bp (18S: PP378514), 1,336 bp (28S: PP378508–PP378513) and 1,539 bp (COI: PP375978–PP375983) were submitted to the GenBank database (Supplementary Table S1).

The ingroup dataset contained sequences of 14 representatives of 16 valid species [34, 67]; no sequences of *Metagonimus ovatus* Yokogawa, 1913 and *Metagonimus minutus* Katsuta, 1932 were available in this study. The isolates for the outgroup were selected according to the phylogeny in Kuzmina *et al*. [50] and Hernández-Orts *et al*. [29]. The alignment was created by concatenating four genetic markers (28S, 18S, COI, and partial ITS1-5.8S-ITS2 ribosomal region) with a total length of 4,858 bp (see Supplementary Table S1 for details). The isolates for each marker were aligned using MAFFT v7.490 with the algorithms E-INS-i for the ribosomal genes and L-INS-i for the mitochondrial gene [45]. Pairwise similarities were calculated as uncorrected *p*-values. The optimal evolution models were estimated separately for the alignment of each marker with ModelFinder using the AICc criterion [36, 43]. Phylogenetic relationships were calculated from the concatenated alignment using the models TVM+F+I+G4 (28S), TN+F+I+G4 (COI, codon1), HKY+F+I (COI, codon2), TIM+F+G4 (COI, codon3), GTR+F+G4 (ITS) and K3Pu+F+I (18S) in IQtree 2.0.5. using ultrafast bootstrapping with 1,000 replicates, Bayesian-like transformation (aBayes) and Shimodaira–Hasegawa approximate likelihood ratio tests (SH-aLRT) [2, 32, 62].

## Results

### *Metagonimus romanicus* (Ciurea, 1915) Ransom, 1920

Syns.: *Loossia romanica* Ciurea, 1915; *Loossia dobrogiensis* Ciurea, 1915; *Loossia parva* Ciurea, 1915.

*Type host*: Domestic dog, *Canis familiaris* L. (Carnivora: Canidae).

*Additional natural definitive hosts* (most records as *M. yokogawai*): (i) *mammals*: European jackal *Canis aureus moreoticus* Saint-Hilaire (Italy [51]); *C. familiaris* (Croatia and Slovenia [6–8, 25]; Russia [37, 38, 85]; Ukraine [23, 39]); red fox *Vulpes vulpes* (L.) (Austria [31]; Serbia [57, 72]; Slovenia [78]; Ukraine [39]) (Carnivora: Canidae); domestic cat *Felis catus* L. (Slovakia [99]; Turkey [16] (as *M. ciureanus*); Ukraine [17, 23, 46, 59]) (Carnivora: Felidae); (ii) *birds*: white-tailed eagle *Haliaeetus albicilla* (L.) (Czech Republic [84]; Romania [15]); black kite *Milvus migrans* (Boddaert) (Accipitriformes: Accipitridae) (Romania [15]); European herring gull *Larus argentatus* Pontoppidan (Charadriiformes: Laridae) (Bulgaria [95]; Ukraine [52, 53]); white stork *Ciconia ciconia* (L.) (Ciconiiformes: Ciconiidae) (Bulgaria [95]); purple heron *Ardea purpurea* L. (Ukraine [10, 86]); black-crowned night-heron *Nycticorax nycticorax* (L.) (Pelecaniformes: Ardeidae) (Ukraine [10, 86]); Dalmatian pelican *Pelecanus crispus* Bruch (Bulgaria [95]; Croatia and Slovenia [6–8, 25]); great white pelican *Pelecanus onocrotalus* L. (Pelecaniformes: Pelecanidae) (Romania [15]); glossy ibis *Plegadis falcinellus* (L.) (Pelecaniformes: Threskiornithidae) (Bulgaria [95]; Croatia and Slovenia [6–8, 25]); pygmy cormorant *Microcarbo pygmaeus* (Pallas) (Romania [15]); great cormorant *Phalacrocorax carbo* (L.) (Suliformes: Phalacrocoracidae) (Poland [44]; Ukraine [10]).

*Experimental definitive* hosts (most records as *M. yokogawai*): (i) *mammals*: Domestic pig *Sus domesticus* (Artiodactyla: Suidae) [13, 15]; European polecat *Mustela putorius* (Carnivora: Mustelidae) (without eggs [15]); golden hamster *Mesocricetus auratus* Waterhouse (Rodentia: Cricetidae) [12] (as *Metagonimus* sp.; present study); (ii) *birds*: Common buzzard *Buteo buteo* (L.); rough-legged buzzard *Buteo lagopus* (Pontoppidan) (Accipitriformes: Accipitridae) [15]; chick *Gallus domesticus* L. (Galliformes: Phasianidae) [12] (present study); long-eared owl *Asio otus* (L.) (Strigiformes: Strigidae) [15].

*Prevalence*: Not reported for adults.

*Site of infection*: Small intestine (mostly middle and posterior third).

*Intensity of infection*: up to 50 in hamsters (experimentally infected with 50 metacercariae; present study); up to 600 in dogs and 112 in cormorants [15]; up to 3,000 in experimentally infected dogs [47].

*Type locality*: Somova, Tulcea County, Romania.

*Distribution and records of adults and metacercariae (indicated by asterisk)* (Fig. 4): Austria [31], Bulgaria* [3, 26, 42, 66, 70, 95], Croatia (Neretva River delta [25]), Czech Republic [84], Hungary* [12, 64], Italy [51], Poland [44], Romania* [13–15], Russia (?) (Rostov Oblast [85]), Serbia* [11, 19, 72], Slovakia* [71, 91, 97–100], Slovenia [78], Turkey [16] (as *M. ciureanus* from Sapanca), Ukraine* [10, 17, 23, 37–39, 46, 59, 86], former Yugoslavia – Croatia and/or Slovenia [6–8].

**Figure 1:**
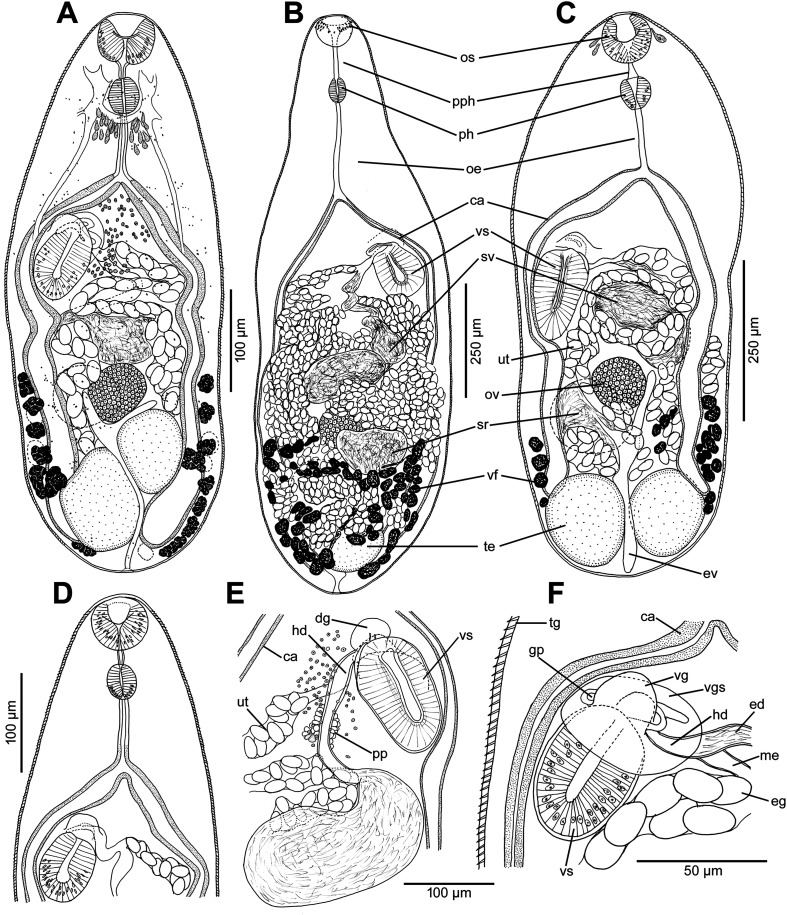
Line drawings of *Metagonimus romanicus* (Ciurea, 1915) (A, B, D–F) and *M. ciureanus* (Witenberg, 1929) (C). **A** – adult from experimentally infected golden hamster (*Mesocricetus auratus*; 8 days post infection – DPI), Hungary, general view, ventral; **B** – syntype from domestic dog (*Canis familiaris*), Somova, Tulcea County, Romania, July 1914 (USNM 1327535), general view, dorsal; **C** – syntype from domestic cat (*Felis catus*), Jaffa, Israel, 1928 (NHMUK 1929.7.24.55–74a); general view, ventral; **D** – adult from golden hamster (8 DPI), anterior part of body with short, but distinct prepharynx; **E** – adult from domestic cat (17 DPI), terminal genitalia, dorsal; note the short hermaphroditic duct and the *pars prostatica*; **F** – adult from golden hamster (8 DPI), terminal genitalia, ventral; note the ventrogenital sac and the ventral gonotyl. Abbreviations: ca – caecum, dg – dorsal gonotyl, ed – ejaculatory duct, eg – egg, ev – excretory vesicle, gp – genital pore, hd – hermaphroditic duct, oe – oesophagus, os – oral sucker, ov – ovary, ph – pharynx, pph – prepharynx, pp – pars prostatica, sr – seminal receptacle, sv – seminal vesicle, te – testes, tg – tegument with spines, ut – uterus, vf – vitelline follicles, vg – ventral gonotyl, vgs – ventrogenital sac, vs – ventral sucker.

**Figure 2:**
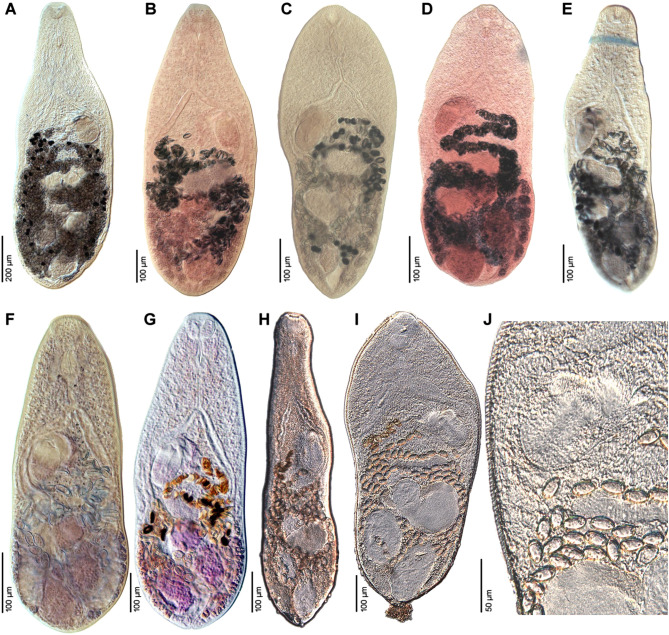
Photomicrographs of *Metagonimus romanicus* (Ciurea, 1915). **A** – syntype from domestic dog (*Canis familiaris*), Somova, Tulcea County, Romania, July 1914 (USNM 1327535); **B** – specimen from experimentally infected cat (*Felis catus*), Slovakia (ESM Z-11235); **C** – specimen from white-tailed eagle (*Haliaeetus albicilla*), Czech Republic (IPCAS D-422/2); **D** – specimen from great cormorant (*Phalacrocorax carbo*), Poland; **E** – voucher from cat, Turkey (USNM 1339602); **F, G** – specimens from experimentally infected (F) golden hamster (*Mesocricetus aureus*) and (G) chick (*Gallus domesticus*), Hungary (IPCAS D-876/1, 2); **H–J** – specimens from experimentally infected golden hamster *in situ*.

*Type material*: Syntypes – USNM 1327535: vial with 20 specimens from dogs in Somova, Romania, collected in 1914; five specimens now stained by the present authors and mounted – Figures 1B, 2A; additional syntypes not studied by present authors: Natural History Museum, Gothenburg, Sweden – MNH GNM 1416, 1417.

*Life cycle*: First intermediate hosts: *Esperiana esperi* (Férussac) and *Microcolpia daudebartii acicularis* (Férussac) (syn. *Fagotia acicularis* [Férussac]) (Caenogastropoda: Melanopsidae) in Poland (Western Polesia), Slovakia and Ukraine [28, 41, 92, 96].

Second intermediate hosts: Broad spectrum of freshwater fish, almost exclusively cypriniforms (Cyprinoidei: Leuciscidae); metacercariae are encysted in scales [12, 15, 19, 42, 49]. Cech *et al*. [12] found metacercariae of *M. romanicus* in six leuciscid species and also in a single European perch (*Perca fluviatilis* L.) (Perciformes: Percidae) in Hungary. In the present study, metacercariae from chub and common nase were used for the experimental infection.

*Phylogenetic relationships*: *Metagonimus romanicus* forms the earliest diverging branch within the genus *Metagonimus* (Fig. 5). Pairwise genetic similarities inferred from the 28S alignment were 0–0.3% within the *M. romanicus* clade and 1.2–3.8% when compared with isolates from East Asia. The similarities calculated from the COI alignment were 0–0.4% and 15.9–20.2%, respectively. To date, one 18S, six 28S and six COI sequences of *M. romanicus* from adult flukes from experimentally infected golden hamsters (see Supplementary Table S1) have been newly generated; another 30 sequences (OQ286071–OQ286088, OQ286093–OQ286097, OQ281688–OQ281703, OQ308609) of *M. romanicus* from the Danube and Lake Balaton (both in Hungary) are currently available in GenBank [12].

#### Redescription (Figs. 1A, 1B, 1D–1F, 2, 3)

**Figure 3:**
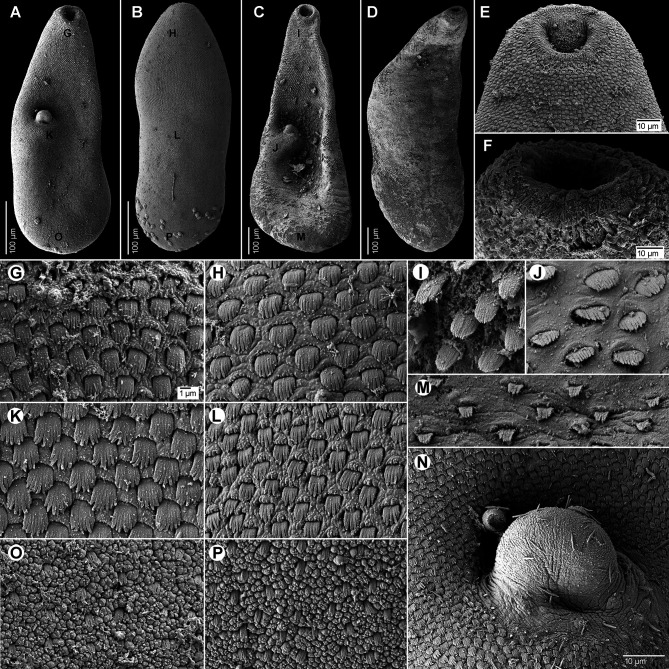
Scanning electron micrographs of *Metagonimus romanicus* (Ciurea, 1915) from experimentally infected golden hamster (*Mesocricetus auratus*; 8 DPI), Hungary (A, B, E, G, H, K, L, N–P) and domestic dog (*Canis domesticus* L.), Romania (syntypes USNM 1327535) (C, D, F, I, J, M). The small letters in A–C indicate the place where the pictures G–P were taken. **A, C** – general view, ventral; **B, D** – general view, dorsal; **E, F** – oral sucker, ventral; **G–M, O, P** – tegumental spines; **N** – ventral gonotyl covering a slit-shaped genital pore. Scale bar in G is also valid for H–M, O and P.

Based on syntypes and vouchers listed above (for measurements – see Table 1): Body small, pyriform, narrowly pyriform to clavate, with maximum width usually in posterior third of body (Figs. 1B, 2A, 3A, 3C). Body covered with tegumental spines except for posterior extremity and around mouth of ventrogenital sac (Figs. 1A, 1B, 2, 3J). Tegument thick, covered with numerous flat, pectinate spines around 1–3 long by 1–4 wide, with 2–9 tooth-like projections (digits) and denser and more numerous anteriorly, become sparse and with fewer digits posteriorly toward end of body (with 6–9 digits anteriorly, 4–8 digits in middle part and with 2–4 digits in posterior part) (Figs. 3G–3N). Several presumably sensory papillae observed on lips of oral sucker (Figs. 3E, 3G). Small dark granules scattered throughout body, especially its anterior half (Fig. 1A). Large gland cells posterior to pharynx (Fig. 1A). Neural ring posterior to pharynx, with main longitudinal cords directed posteriorly to cross caeca at anterior level of ventral sucker (Fig. 1A).

**Table 1:** Comparative measurements of *Metagonimus romanicus* (Ciurea, 1915) and *M. ciureanus* (Witenberg, 1929).

Species (revised identification)	*M. romanicus*	*M. romanicus*	*M. romanicus*	*M. romanicus*	*M. romanicus*	*M. romanicus*	*M. romanicus*	*M. ciureanus*	*M. ciureanus*	*M. ciureanus*
Species (original identification)	*Loossia romanica*	*M. romanicus*	*M. romanicus*	*M. romanicus*	*M. romanicus*	*M. yokogawai*	*M. yokogawai*	*Dexiogonimus ciureanus*	*D. ciureanus*	*D. ciureanus*
Host	*Canis familiaris*	*Canis familiaris*	*Canis familiaris*	*Canis familiaris*	*Mesocricetus auratus*	*Felis catus*	*Haliaeetus albicilla*	*Felis catus*	*Felis catus*	*Vulpes corsac*, *Canis aureus*, *Felis chaus*
Country	Romania	Romania	Romania	Georgia	Hungary	Slovakia	Czechia	Israel	Israel	Georgia
Collection No.	N/A	USNM 1327535	USNM 1341217	N/A	IPCAS D–876/1	ESM Z–11235	IPCAS D–422/2	USNM 1339602	NHMUK 1983.1.11.2, 2000.4.10.103	N/A
Reference	Ciurea (1915)	This study	This study	Isaychikov (1925)	This study	This study	This study	Witenberg (1929)	This study	Rodonaya (1967)
Remark	Original description	Syntypes (*n* = 5)	Vouchers (*n* = 2)	Vouchers (*n* = not given)	Experimental host (8 DPI; *n* = 8)	Experimental host (17 DPI; *n* = 8)	*n* = 3	Original description	Syntypes (*n* = 8)	Vouchers (*n* = not given)
Body length (mm)	0.60–1.56	1.01–1.24	0.98–1.18	1.38–1.86	0.36–0.62	0.86–1.11	0.69–0.82	0.70–1.30	0.71–1.08	1.06–1.36
Body width	400–540	396–440	404–461	530–795	160–242	266–423	241–283	300–700	276–455	340–540
Forebody length	N/A	381–596	429–534	N/A	179–280	317–495	312–413	N/A	327–505	N/A
Forebody (% of total length)	N/A	38–52%	43–45%	59%	42–57%	41–49%	45–51%	N/A	44–50%	N/A
Oral sucker length	81	60–84	64–80	74–106	40–62	59–78	58–62	N/A	52–80	60–82
Oral sucker width	110	78–86	78–83	95–117	45–64	67–81	65–71	50–90	64–82	N/A
Ventral sucker length	90–165	94–135	131–134	159–233	46–80	107–151	77–188	N/A	113–200	N/A
Ventral sucker width	96–114	74–97	88–90	72–127	33–58	65–89	57–124	N/A	62–104	N/A
O:V sucker width ratio	N/A	N/A	N/A	N/A	N/A	N/A	N/A	N/A	N/A	N/A
Ventral sucker position	N/A	N/A	N/A	519–689	N/A	N/A	N/A	N/A	N/A	N/A
Prepharynx length	22–74	32–69	29–35	N/A	6–22	10–28	15–37	20–60	11–49	20–65
Pharynx length	50–63	51–61	48–49	53–74	31–47	33–51	43–47	30–50	36–60	N/A
Pharynx width	30–44	34–42	36–40	42–53	22–35	33–45	31–37	N/A	33–44	40–49
Oesophagus length	86–143	109–243	123–161	180–254	37–80	113–164	68–130	60–90	64–144	100–190
Dorsal gonotyl length	N/A	27–32	28–32	N/A	17–21	22–37	19–31	N/A	37	N/A
Dorsal gonotyl width	N/A	36–45	40–45	N/A	23–31	32–52	27–34	N/A	31	N/A
Ventral gonotyl length	N/A	42–82	55–71	N/A	17–30	43–73	29–36	N/A	28	N/A
Ventral gonotyl width	N/A	37–46	30–54	N/A	23–37	26–52	24–36	N/A	20	N/A
Position of testes	N/A	Diagonal	Diagonal	N/A	Diagonal	Diagonal	Diagonal	N/A	Vertical	N/A
Testis length (anterior)	N/A	104–129	128–141	180–254	53–96	118–170	78–191	180	98–141	140–180
Testis width (anterior)	N/A	79–106	105–106	127–244	48–63	101–146	60–170	90	68–116	N/A
Testis length (posterior)	N/A	123–147	146–157	201–233	56–101	116–162	77–88	N/A	96–175	140–180
Testis width (posterior)	N/A	83–110	111–126	159–276	48–74	103–145	55–64	N/A	73–97	N/A
Seminal vesicle length	N/A	103–142	108–159	N/A	65–104	154–236	105–133	300	119–164	200–350
Seminal vesicle width	N/A	73–92	52–66	N/A	43–63	69–110	58–71	N/A	47–79	N/A
Ovary length	N/A	108–132	118–134	148–191	44–76	93–139	60–125	130	66–100	100–120
Ovary width	N/A	68–104	86–97	117–201	38–55	80–101	52–125	70	55–100	N/A
Seminal receptacle length	N/A	91–201	83–123	212–265	49–64	104–161	94–101	Larger than ovary	127–148	100–150
Seminal receptacle width	N/A	61–105	63–111	212–244	39–52	70–113	65–66	N/A	59–109	N/A
Anterior extent of vitellarium	N/A	To ovary	To ovary	To ovary	To ovary	To ovary	To ovary	To ovary	To ovary	N/A
Posterior extent of vitellarium	N/A	To posterior testis	To posterior testis	To posterior testis	To posterior testis	To posterior testis	To posterior testis	To middle part of testes	To middle part of testes	N/A
Uterine region length	N/A	503–612	554–611	N/A	157–321	412–605	323–344	N/A	314–543	N/A
Anterior extent of uterus	N/A	To ventral sucker	To ventral sucker	To ventral sucker	To ventral sucker	To ventral sucker	To ventral sucker	To ventral sucker	To ventral sucker	N/A
Posterior extent of uterus	N/A	To posterior testis	To posterior testis	To posterior testis	To anterior/posterior testis	To posterior testis	To posterior testis	To anterior part of testis	To anterior part of testis	N/A
Distance of uterus from anterior end	N/A	377–552	374–504	N/A	170–248	313–456	261–375	N/A	279–452	N/A
Egg length	N/A	23–26	23–26	23–27	23–27	23–26	23–27	25–28	25–28	25–26
Egg width	N/A	13–15	13–16	14–16	13–16	14–17	14–16	15	14–17	13–16

N/A – data not available.

Oral sucker subterminal, spherical, without circumoral spines or posterior appendage (Figs. 1A, 1D, 3E, 3F). Prepharynx variable in length, almost always present, straight or slightly sinuous (Figs. 1A, 1B, 1D, 2A–2I); pharynx oval, strongly muscular (Figs. 1A, 1D, 2A–2I). Oesophagus straight, much longer than prepharynx (Figs. 1A, 1B, 1D, 2A–2I). Intestinal bifurcation pre-equatorial; intestinal caeca thick-walled (Figs. 1A, 1F, 2A–2I), ventral, narrow and long, slightly sinuous, reaching near posterior extremity (to posterior level of posterior testis), slightly bent inwards (medially) in their terminal part (Figs. 1A, 2A–2I).

Ventrogenital complex situated obliquely (axis inclined anterosinistrally), median to dextral caecum, posterolateral to caecal bifurcation, equatorial to slightly pre- or postequatorial (Figs. 1A, 1B, 1D–1F, 2). Ventral sucker conspicuously dextral, slightly obliquely embedded in parenchyma, protruding into ventrogenital sac (Figs. 1F, 2J). Sucker strongly muscular, elongate to ovoid, larger than oral sucker (Figs. 1A, 1B, 1D–1F, 2), with narrow cavity opening anteromedially into ventrogenital sac (Figs. 1F, 2J) and inner margin formed by strong longitudinal muscles and prominent nuclei in posterior half (Figs. 1A, 1F). Ventrogenital sac large, thick-walled, widely oval (Figs. 1F, 2J). Two gonotyls on walls of ventrogenital sac; ventral gonotyl prominent, large, flap-like, partly overlapping (closing) genital pore (Figs. 1F, 2J, 3A, 3N); dorsal gonotyl smaller, fleshy, wider than long, situated anterosinistral to ventral gonotyl (Fig. 1E). Genital pore slit-shaped, oblique, slightly curved, anterosinistral to ventral sucker (Figs. 1F, 2J, 3N).

Testes two, on ventral side of body, smooth, widely oval to almost spherical, oblique, situated close to each other (Figs. 1A, 2A–2I), with posterior (dextral) testis near posterior extremity (Figs. 1A, 1B); testes of similar size or anterior (sinistral) testis smaller than posterior (dextral) testis (Fig. 1A). Seminal vesicle voluminous, posteromedian to ventral sucker, anterior to ovary (Figs. 1A, 1B, 1E). Cirrus sac and cirrus absent. Pars prostatica short, surrounded by large prostatic cells (Fig. 1E). Ejaculatory duct short, thick-walled, slightly sinuous, joining metraterm to form short, thick-walled hermaphroditic duct (called genital atrium by Shimazu and Kino (2015) [81]), opening into ventrogenital sac (Figs. 1E, 1F). Small round gland cells scattered alongside ejaculatory duct, metraterm and hermaphroditic duct (Figs. 1A, 1E).

Ovary compact, smooth, widely oval to spherical, median to slightly submedian, between seminal vesicle and anterior testis (Figs. 1A, 2A–2I). Seminal receptacle dorsal, voluminous, transversely oval, submedian (dextral), posterolateral to almost lateral to ovary (Fig. 1B), opening to oviduct on dorsal side of ovary. Laurer’s canal short, thick-walled, anterolateral to ovary, coiled, on dorsal side, but not opening outside, containing sperm.

Vitellarium formed by relatively few follicles in posterior fourth or third of body (Figs. 1A, 1B, 2A–2I). On ventral side, vitelline follicles form short, narrow band between preovarian level and posterior extremity (Fig. 1A). On dorsal side, vitelline follicles scattered throughout posterior fourth of body between seminal receptacle and posterior extremity (Fig. 1B). Common vitelline ducts slightly sinuous, almost horizontal, posterior to ovary and anterior part of seminal receptacle, joined medially.

Uterus tubular, forming several loops on ventral and dorsal side between ventral sucker and posterior testes (Figs. 1A, 2A–2I), but never reaching posterior to posterior testes (Figs. 1B, 2A–2I). Uterine loops partly overlap seminal receptacle (Fig. 1A) and anterior half of posterior testis on ventral side, and anterior half of ovary and whole anterior testis on dorsal side (Fig. 1B). Metraterm short, thick-walled (Figs. 1E, 1F), joining ejaculatory duct to form short hermaphroditic duct anteromedian to ventral sucker (Fig. 1E, 1F). Eggs numerous (Figs. 1B, 2J), thick-walled, operculate.

Excretory vesicle ventral, slightly asymmetrically Y-shaped, with sinistral arm reaching to level of ovary and dextral arm reaching to seminal receptacle (Fig. 1A). Excretory pore posterodorsal.

#### Remarks

This species was described by Ciurea (1915) [13] as *Loossia romanica* from a domestic dog from the Danube Delta in Romania as the type species of *Loossia* Ciurea, 1915, together with *L. parva* Ciurea, 1915 from domestic cats and *L. dobrogiensis* Ciurea, 1915 from pelicans. However, Ransom (1920) [77] considered *Loossia* to be a junior synonym of *Metagonimus* and synonymised all species with *M. yokogawai* (Katsurada, 1912), the type species of the genus and the most important zoonotic species of the genus described in Japan. Ciurea (1924) [14] accepted the synonymy of *Loossia* with *Metagonimus*, but disagreed with the synonymisation of its three species with *M. yokogawai*. Similarly as Ransom (1920) [77], *L. romanicus*, *L. parva* and *L. dobrogiensis* are considered by the present authors conspecific and members of *Metagonimus*, but distinct from *M. yokogawai*.

Isaychikov (1925) [37] found *Metagonimus* trematodes in domestic dogs in Crimea and accepted the generic assignment of *L. romanica* to *Metagonimus* (although misspelled as *M. romanica*). In contrast to Ransom [77], Isaychikov [37] considered this species to be valid and not a synonym of the Asian *M. yokogawai*, due to the markedly different geographical distribution of the two taxa and the considerable differences in the molluscan and fish fauna in East Asia on the one hand, and Europe on the other. However, later authors, including Morozov (1952) [65], identified European specimens as *M. yokogawai* [10, 46, 48, 49, 84, 86].

The identification of *Metagonimus* metacercariae in scales of cyprinoids and other freshwater fish is similar. Prettenhoffer (1930) [75] and Ciurea (1933) [15] identified the metacercariae found in cyprinoids in Hungary and Romania as *M. romanicus*, while other authors identified them as *M. yokogawai* [42, 49, 64, 76, 91, 101].

Cech *et al*. [12] demonstrated that these trematodes from Danube fish are molecularly distinct from *M. yokogawai* and other Asian species, and suggested that they may belong to *M. romanicus*, but did not compare their specimens morphologically with those of *M. romanicus*. The present study confirms the conspecificity of these trematodes with *M. romanicus*, which was described in Romania, and provides detailed morphological characteristics and sequence data to distinguish it from the congeneric species, including *M. yokogawai*.

*Metagonimus romanicus* differs from *M. yokogawai*, redescribed by Shimazu and Kino [81], by the presence of a prepharynx and a long oesophagus in *M. romanicus* (the prepharynx is absent in *M. yokogawai* and the oesophagus is short), the position of the ventral sucker (equatorial versus clearly pre-equatorial in the latter species) and the ovary (at 2/3 of the body length versus almost equatorial in the latter species), the anterior extension of the vitelline follicles (limited to the posterior third of the body in *M. romanicus*, whereas in *M. yokogawai* they extend almost to the middle of the body) and the posterior extension of the uterus (the uterus extends almost to the posterior margin of the posterior testis in *M. romanicus*, whereas in *M. yokogawai* it only reaches the anterior margin of the posterior testis) (see Figs. 1A, 1B, 1D–1F, 2A–2G, 3, 6C in this paper and Figs. 1–6 in [81]). *Metagonimus romanicus* differs from all but one (*M. ciureanus*) known species of the genus *Metagonimus* by the presence of a prepharynx [67, 81, 82, 94].

López-Neyra and Guevara Pozo (1932) [56] found small operculated eggs, presumably belonging to heterophyid trematodes, in a single stool sample from a 23-year-old man from Granada, Spain. They identified these eggs as most likely belonging to *M. romanicus* based on their size (26.6–29.0 × 15–20 μm; mean 27.4 × 18.0 μm), morphology and colour. However, the eggs of heterophyids are very similar, and reliable species identification based on morphology alone is not possible [18]. Furthermore, this Spanish patient never ate raw or smoked fish, not even freshwater fish. After his treatment, no adult specimens were excreted that would have allowed an accurate identification. Later, López-Neyra (1941) [55] reported this finding under the name *M. yokogawai* and listed *M. romanicus* (misspelled as *Lossia romanica*) as its synonym. This author also reported the occurrence of unidentified heterophyid metacercariae in the scales of goldfish (*Carassius auratus* L.) from Granada [55].

*Metagonimus romanicus* is a widespread intestinal parasite of a variety of piscivorous mammals (four species, including three other experimental hosts) and birds (no less than 12 bird species have been identified as hosts for *M. romanicus*) in central and southeastern Europe, especially in the lower reaches of rivers flowing into the Black Sea (mainly the Danube, Dniester and Dnieper), while it is apparently absent from the upper reaches of the Dnieper and Danube [20, 49]. This distribution follows the distribution of the first intermediate hosts, *Esperiana esperi* and *Microcolpia daudebartii acicularis* [27, 89, 90]. The most suitable definitive hosts are dogs, cats and probably also domestic pigs, in which the trematodes survive for up to 86 days [15].

Based on this distribution area, we assume that the specimens from dogs in the Rostov region in Russia reported by Skryabin and Lindtrop [85] could also belong to *M. romanicus*, but no morphological description or voucher specimens are available. A single immature specimen identified as *M. yokogawai* was also found in a great cormorant from northern Poland [44], but this appears to be an accidental record of the parasite being introduced into Poland by a migratory definitive host.

Specimens from domestic cats from Sapanca in Turkey [16] (USNM 1339602), identified as *M. ciureanus*, have oblique rather than symmetrical testes and are morphologically indistinguishable from *M. romanicus*. These trematodes represent the southernmost record of *M. romanicus*. However, it is not clear whether the distribution range of *M. romanicus* overlaps with that of *M. ciureanus*, which occurs in Transcaucasia and the Middle East (see below).

### *Metagonimus ciureanus* (Witenberg, 1929) Price, 1931

Syn. *Dexiogonimus ciureanus* Witenberg, 1929.

*Type host*: Domestic cat *Felis catus* L. (Carnivora: Felidae); Witenberg (1929) [94] did not explicitly designated the type host, but syntypes were found in domestic cat.

*Additional natural definitive hosts:* (i) *mammals*: golden jackal *Canis aureus* L. (Georgia [79]; Iran [61]); *C. familiaris* (Georgia [21, 48]; Iraq [1]) (Carnivora: Canidae); domestic cat *Felis catus* L. (Israel [94], Jordan [40]); jungle cat *Felis chaus* Schreber (Georgia [79]); corsac fox *Vulpes corsac* L. (Georgia [79]); red fox *Vulpes vulpes* (L.) (Iran [61]) (Carnivora: Canidae); (ii) *birds*: *Larus* sp. (Charadriiformes: Laridae) (Israel [94]), great cormorant *Phalacrocorax carbo* (L.) (Suliformes: Phalacrocoracidae) (Azerbaijan [58]; Israel [22]).

*Site of infection*: Small intestine.

*Prevalence*: Two infected cats of 123 examined in Jordan [40].

*Intensity of infection*: 5–200 per host [79].

*Type locality*: Israel (precise locality not specified; syntypes were found in Jaffa, Jerusalem and the neighbourhood of the Lake Tiberias (Sea of Galilee).

**Figure 4:**
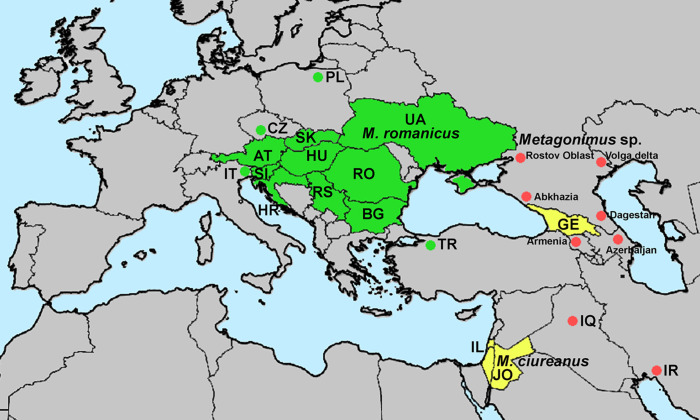
Geographical distribution of *Metagonimus romanicus* (Ciurea, 1915) in green and *M. ciureanus* (Witenberg, 1929) in yellow. Records that could not be reliably identified to species level in red.

*Distribution and records (records of metacercariae indicated by an asterisk; uncertain records marked with a question mark)* (Fig. 4): Abkhazia (?) [4]; Armenia (?) [10], Azerbaijan (?) [58, 60]; Georgia [21, 48, 68, 79]; Iran (?) [61] (Khuzestan); Iraq (?) [1]; Israel* [22, 94]; Jordan [40]; Russia* (?) [80] (Astrakhan Oblast), [24] (Dagestan).

*Type material*: Syntypes – NHMUK 1929.7.24. 55*–*74: two vials with 57 specimens from cat in Jaffa, currently part of Tel Aviv, Israel, collected in 1928; six of these specimens were stained by the present authors and mounted as permanent preparations (Figs. 1C, 6A); NHMUK 2000.4.10.103: one vial with six specimens from experimentally infected cat in Jerusalem, 1927; two of these specimens were stained and mounted by the present authors; additional syntypes not studied by the present authors: Natural History Museum, Berlin, Germany – ZMB 5274-E (vial labelled “unspec. Type” from cat, Jerusalem); Helminthological Laboratory of the Russian Academy of Sciences, Moscow, Russia – GELAN 13628 (two syntypes); USNM 1330229 (vial with syntype specimens from cat, Tiberias, Sea of Galilee, Israel).

*Life cycle*: First intermediate host is not known, but several reports of “*Cercaria metagonimus yokogawai*” or “*Cercaria metagonimus* sp.” are known from *Melanopsis buccinoidea* (Olivier) (Caenogastropoda: Melanopsidae) in Georgia and other species of *Melanopsis* Férussac in Azerbaijan (erroneously reported as *Melanopsis praemorsa* [L.]) [27, 60, 68]. Freshwater fish serve as second intermediate hosts. Witenberg [94] found metacercariae, which presumably belong to *M. ciureanus*, in the cichlids *Tilapia simonis* (= *Tristamella simonis* [Günther]) and *Tilapia galilea* (= *Sarotherodon galilaeus* [L.]) (Cichliformes), which are the most important intermediate fish hosts, cyprinids *Barbus canus* (= *Carasobarbus canis* [Valenciennes]) (Cypriniformes), *Discognathus* sp. (= *Garra* sp.), mugilids *Mugil cephalus* L., *M. capito* (= *Chelon ramada* [Risso]) (Mugiliformes) and carangids *Lichia glauca* (= *Trachinotus ovatus* [L.]) (Carangiformes). However, some of these records need to be verified, as other heterophyids (*e.g.*, *Heterophyes heterophyes* Katsurada, 1912) can also infect fish such as mugilids or carangids.

**Figure 5:**
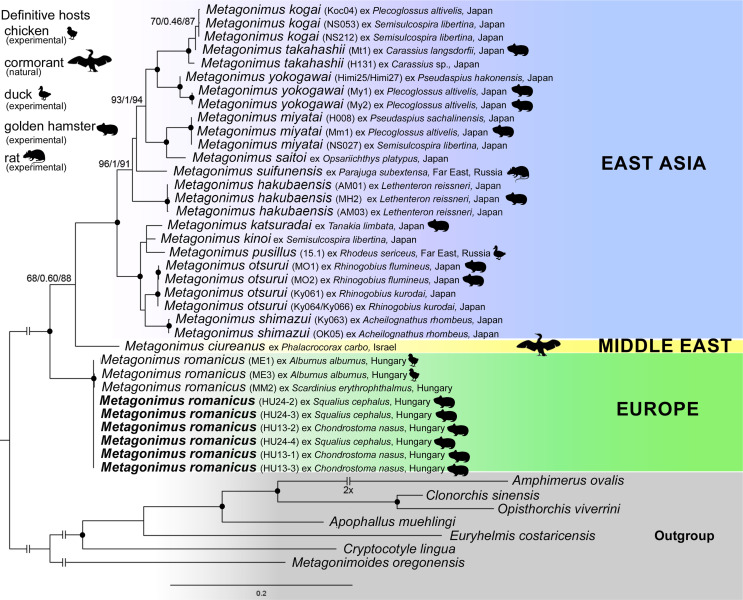
Phylogenetic relationships within the genus *Metagonimus* calculated by ultrafast bootstrapping (UFBoot) based on concatenated alignment of four genetic markers (18S RNA, 28S rRNA and COI genes, and ITS region). Nodal support is indicated by black dots when SH-aLRT ≥ 80, aBayes ≥ 0.95, UFBoot ≥ 95, or indicated as values near the nodes when the UFBoot value exceeds 80. The bar shows the number of substitutions per site. For more data on sequenced samples – see Supplementary Table S1.

*Phylogenetic relationships*: To date, only one sequence of the nearly complete 18S rRNA gene and complete ITS ribosomal region of *M. ciureanus* from great cormorant in Israel is available (AY245702; Dzikowski *et al.* 2004 [22]). This isolate is nested in a strongly supported clade of the genus *Metagonimus* (see Fig. 5 and [12]). Its relatively long lineage clustered with East Asian isolates in the present analyses, whereas it forms a sister taxon to the *M. romanicus* clade in Cech *et al*. [12]. As supports for this node in both analyses are low, the phylogenetic position of *M. ciureanus* within the genus *Metagonimus* remains unclear.

#### Redescription (Figs. 1C, 6A–6B, 7)

**Figure 6:**
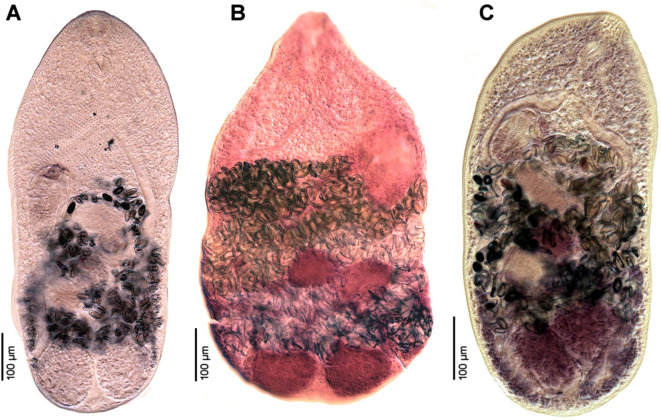
Photomicrographs of *Metagonimus ciureanus* (Witenberg, 1929) (A, B) and *M. yokogawai* (Katsurada, 1912) (C). **A** – syntype from cat (*Felis catus*), Israel (NHMUK 1929.7.24.55–74); **B** – specimen from cat, Jordan (NHMUK 1983.1.11.2); **C** – specimen from experimentally infected golden hamster (*Mesocricetus aureus*), Japan (IPCAS D-422/1).

**Figure 7:**
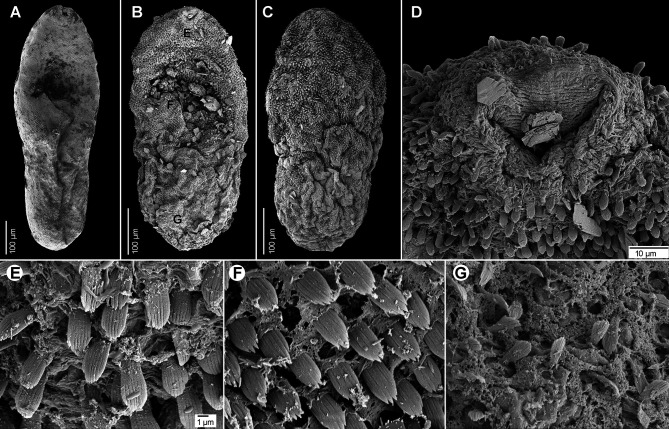
Scanning electron micrographs of *Metagonimus ciureanus* (Witenberg, 1929) from the domestic cat (*Felis catus* L.), Israel (syntype: A – NHMUK 2000.4.10.103; B-G – NHMUK 1929.7.24.55-74). The small letters in B indicate the place where the pictures E–G were taken.

Based on syntypes and vouchers listed above (for measurements – see Table 1): Body small, pyriform to narrowly pyriform, with maximum width between first and second third of body (Figs. 1C, 6A, 6B, 7). Body covered with tegumental spines except for posterior extremity and around mouth of ventrogenital sac (Fig. 7). Tegument thick, covered with numerous flat, pectinate spines around 1–3 by 1–4 wide, with 2–10 tooth-like projections (digits) and denser and more numerous anteriorly, become sparse and with fewer digits posteriorly toward end of the body (with 5–10 digits anteriorly, 4–9 digits in middle part and with 3–5 digits in posterior part) (Figs. 7E–7G). Several presumably sensory papillae observed on lips of oral sucker (not visible in Fig. 7D due to damaged tegument). Small dark granules and neural ring with main longitudinal cords not seen in stained and mounted syntypes.

Oral sucker subterminal, spherical, without circumoral spines or posterior appendage (Figs. 1C, 6A, 6B, 7D). Prepharynx short, straight (Figs. 1C, 6A, 6B); pharynx oval, strongly muscular (Figs. 1C, 6A, 6B). Oesophagus straight, much longer than prepharynx (Figs. 1C, 6A, 6B). Intestinal bifurcation pre-equatorial; intestinal caeca thick-walled, ventral, wide, slightly sinuous, not reaching posterior extremity, ending at anterior portion of testis, slightly bent outwards (externally) in their terminal part (Figs. 1C, 6A, 6B).

Ventrogenital complex similar to that of *M. romanicus* (see above), situated obliquely (axis inclined anterosinistrally), median to dextral caecum, posterolateral to caecal bifurcation, pre-equatorial to almost equatorial (Figs. 1C, 6A, 6B). Ventral sucker conspicuously dextral, slightly obliquely embedded in parenchyma, protruding into ventrogenital sac. Sucker strongly muscular, elongate to ovoid, larger than oral sucker, with narrow cavity opening anteromedially into ventrogenital sac and inner margin formed by strong longitudinal muscles and prominent nuclei in posterior half (Fig. 1C). Ventrogenital sac not observed in stained and mounted syntypes. Two gonotyls on walls of ventrogenital sac; ventral gonotyl prominent, large, flap-like, partly overlapping (closing) genital pore (Fig. 1C); dorsal gonotyl smaller, fleshy, wider than long, situated anterosinistral to ventral gonotyl (Fig. 1C). Genital pore slit-shaped, oblique, slightly curved, anterosinistral to ventral sucker.

Testes double, on ventral side of body, smooth, widely oval to almost spherical, symmetrical, situated close to each other near posterior extremity, similar in size (Figs. 1C, 6A, 6B). Seminal vesicle voluminous, transversely oval, posteromedian to ventral sucker, anterior to ovary (Fig. 1C). Cirrus sac and cirrus absent. Pars prostatica not observed. Ejaculatory duct short, thick-walled, slightly sinuous, joining metraterm to form short, thick-walled hermaphroditic duct (called genital atrium by Shimazu and Kino [81]), opening into ventrogenital sac (Fig. 1C).

Ovary compact, smooth, spherical to subspherical, median to slightly submedian, between seminal vesicle and testes (Figs. 1C, 6A, 6B). Seminal receptacle dorsal, voluminous, transversely oval, submedian (dextral), posterolateral to ovary (Fig. 1C). Laurer’s canal not observed.

Vitellarium formed by relatively few follicles on dorsal and lateral sides of body in posterior third of body (Fig. 1C). On ventral side, vitelline follicles form short, narrow band between ovary and testes (Fig. 1C). On dorsal side, vitelline follicles scattered throughout posterior fourth of body between seminal receptacle and testes.

Uterus tubular, forming several loops on ventral and dorsal side between ventral sucker and anterior portion of testes, never reaching to posterior extremity (Figs. 1C, 6A, 6B). Uterine loops partly overlap seminal receptacle and anterior half of testes on dorsal side. Metraterm short, thick-walled, joining ejaculatory duct to form short hermaphroditic duct anteromedian to ventral sucker (Fig. 1C). Eggs numerous, thick-walled, operculate.

Excretory vesicle ventral, slightly asymmetrically Y-shaped, with sinistral arm reaching to level of ovary and dextral arm reaching to seminal receptacle (Fig. 1C). Excretory pore posterodorsal.

#### Remarks

This species was described by Witenberg [94] as *Dexiogonimus ciureanus* from domestic cats and dogs in what was then Palestine (now Israel). The type locality was not given, but most specimens were found in the vicinity of the Lake Tiberias (Sea of Galilee). However, the syntypes studied here were found in Jaffa, and Jerusalem is indicated on the labels of the type material from the NHMUK and the ZMB. It is the only species of the genus *Dexiogonimus* Witenberg, 1929 and differs from *Metagonimus* only in the symmetrical position of the testes, in contrast to the oblique testes of *Metagonimus* species. Pearson (2008) [73] listed *Dexiogonimus* among the synonyms of *Metagonimus*, and molecular analyses by Cech *et al*. [12] and in the present study support this synonymy [22].

Examination of the type specimens of *M. ciureanus* confirmed the validity of the species, which is unique among all *Metagonimus* species in having symmetrical testes. Otherwise, *M. ciureanus* is very similar in morphology and dimensions to *M. romanicus*, but differs in body shape (maximum width between the first and second thirds of the body compared to the posterior third of the body in *M. romanicus*; Figs. 1A–1C, 2A–2D, 3A–3D) and shorter caeca (which in *M. ciureanus* extend only to the anterior margin of the testes, whereas in *M. romanicus* they terminate near the posterior end; Figs. 1A, 1C).

*Metagonimus ciureanus* is a rare species that has so far only been confirmed from Israel, Jordan, and Georgia (Fig. 4). However, it is possible that it also occurs in neighbouring countries, such as Abkhazia, Armenia, Azerbaijan, Iran, Iraq, and probably also in Russia (Astrakhan Oblast, Dagestan), where “*M. yokogawai*” has been reported, but detailed morphological descriptions or voucher material are not available for examination. The life cycle of *M. ciureanus* is not fully known, but *Melanopsis buccinoidea* and other species of *Melanopsis* are most likely its first intermediate host (misidentified as *M. praemorsa*) [60, 68]. These snails are distributed in the Middle East as far as Transcaucasia [27]. Several species of freshwater fish serve as second intermediate hosts, as shown by Witenberg [94], who found metacercariae of presumably the same species in a variety of fish from different groups (see Taxonomic summary).

## Discussion

### Systematics

The present study confirms the results of Cech *et al*. [12], who provided molecular evidence that the metacercariae in the scales of cyprinoids in Central Europe do not belong to the Asian zoonotic species *Metagonimus yokogawai*, which does not occur in Europe. Since these authors did not provide detailed information on adults of this species obtained from experimentally infected hosts (hamsters and chicks), we took the opportunity to compare new, properly fixed adults with the type and voucher specimens of *M. romanicus* deposited in museums. Although *M. romanicus* was described more than 100 years ago, the type material is in good condition, so that a comparison of the morphology with our new material from the Hungarian Danube was possible (Figs. 1, 2, 3).

Although *M. romanicus* was little known and neglected for more than 90 years, especially because of its erroneous identification as *M. yokogawai*, it is indeed a common parasite in Central and Eastern Europe, with most records coming from the middle and lower reaches of the Danube, Dniester and Dnieper rivers. Adults have been found in four mammal and 12 bird species from 14 European countries, with the exception of some experimental hosts (three mammal species and three birds) (Fig. 4). Metacercariae of *M. romanicus* are very common in the scales of numerous cyprinoids and other freshwater fish, especially in the middle and lower section of the Danube [19, 42, 49].

In contrast, *M. ciureanus* is a poorly known species that has only rarely been reported from the Middle East and Transcaucasia (Fig. 4) [22, 40, 79, 94]. The only information on fish intermediate hosts of *M. ciureanus* comes from Witenberg [94], but it is questionable whether metacercariae of this trematode have actually been found in all listed fish, especially in carangids and mugilids, which may serve as intermediate hosts for other heterophyids, such as *Ascocotyle longa* (Ransom, 1920) [29].

The long-standing confusion of *M. romanicus* with *M. yokogawai* is rather surprising. It seems that Ransom [77], who was the first to propose the synonymy of *M. romanicus* with *M. yokogawai*, and other authors who followed this synonymy, did not examine the morphology of the former species, and relied only on the superficial similarity of the two taxa. Moreover, with the exception of Isaychikov [37] and Prettenhoffer [75], they did not take into account the widely separated distribution ranges and the different spectrum of snail and fish intermediate hosts of these two species.

Fortunately, both Ciurea [13] and Witenberg [94] deposited their type material in several collections, so it has been possible to redescribe these species and confirm their validity on the basis of their type specimens. However, it is not possible to verify the species identification of numerous specimens, as no voucher specimens or morphological descriptions with illustrations are available. The lack of voucher specimens in faunistic surveys has been a regrettable practice for decades, and the deposition of voucher specimens is still not common in current ecological and other studies, although the importance of museum specimens has been repeatedly confirmed [30].

*Metagonimus romanicus* differs from all Asian species, including *M. yokogawai*, in several morphological characters and most closely resembles *M. ciureanus*, which is also redescribed in the present paper. Molecular data clearly distinguish *M. romanicus* and *M. ciureanus* from each other and from East Asian species (Fig. 5). However, their relationships remain unclear, as *M. romanicus* as the earliest branch of the genus is poorly supported in the present work and the two species are considered closely related by Cech *et al*. [12]. Nevertheless, there is no reason to resurrect *Loossia* to accommodate *M. romanicus* and *M. ciureanus*, because all species currently belonging to *Metagonimus* are similar in their overall morphology.

### Zoonotic potential

Small intestinal flukes, especially those of the family Heterophyidae, are causative agents of human disease in Asia [33, 35]. Five species of *Metagonimus* have been confirmed as human parasites, almost exclusively in East Asia, whereas no human cases are known from the western Palaearctic region. The zoonotic significance of *M. romanicus* and *M. ciureanus* is not clear, but appears to be low in Europe. There is a report of heterophyid eggs in a human stool sample in Spain that were identified as *M. romanicus* based on their size and colour [56], but this report is doubtful because the eggs of heterophyid and opisthorchiid trematodes are similar and cannot be distinguished under the light microscope [18]. Heterophyid trematodes, including *M. romanicus*, survive for a relatively short time in the definitive host (a few weeks [15]), which means that they can only survive for a short time in humans and do not usually cause serious medical problems [34]. In addition, Massoud *et al*. (1981) [61] reported that 8% of human stool samples in Khuzestan (Iran) were infected with unidentified heterophyid eggs; they also found trematodes identified as *M. yokogawai* and other heterophyids in carnivores.

Human infection with *Metagonimus* trematodes is also unlikely in Europe, as raw, whole fish with scales are generally not consumed. Accidental infection of fishermen, who can become infected with metacercariae in the scales when cleaning fish, is more likely, but has never been reported. Remains of cyprinoids with scales infected with metacercariae of *Metagonimus* spp. may also be an important source of natural infection for dogs, cats, other carnivores and piscivorous birds, as previous studies have shown (see above).

The snail intermediate hosts appear to be a key factor limiting the spread of these trematodes. The first snail intermediate host of *M. romanicus* occurs only in Europe, with the easternmost distribution in Ukraine and southern Russia [27]. Potential snail hosts of *M. ciureanus* are exclusively distributed in the Middle East up to Iran and Transcaucasia. Adults may be transported by migratory or mobile definitive hosts outside the range of the snail intermediate hosts, *e.g*., in eastern Poland and the Volga Delta [44, 80], but the life cycle may not be completed in these regions.

## Supplementary material

The Supplementary materials of this article are available at https://www.parasite-journal.org/10.1051/parasite/2024016/olm. *Supplementary Table S1*: List of sequences of *Metagonimus* species used in phylogenetic analyses.
