# Supplementary material for: Small intestinal flukes of the genus Metagonimus (Digenea: Heterophyidae) in Europe and the Middle East: A review of parasites with zoonotic potential
Source: Parasite. 2024 Mar 29;31:20. doi: 10.1051/parasite/2024016 (PMC10979786; doi:10.1051/parasite/2024016)
Supplement: Supplementary file 1 — Supplementary Table S1: List of sequences of Metagonimus species used in phylogenetic analyses. [file parasite-31-20-s1.pdf]

Supplementary Table S1. List of sequences of *Metagonimus* species used in phylogenetic analyses.

Species (isolate)	Stage	Experimental host	Natural host	Locality	18S rDNA	28S rDNA	ITS1+2	COI	Reference
*M. ciureanus**	adult	N/A	*Phalacrocorax carbo*	Israel	AY245702	N/A	AY245702	N/A	Dzikowski et al. 2004
M. hakubaensis (AM01)	mtc.	N/A	*Lethenteron reissneri*	Japan, Towada	N/A	LC666761	N/A	LC666711	Nakao et al. 2022
M. hakubaensis (AM03)	mtc.	N/A	*Lethenteron reissneri*	Japan, Towada	N/A	LC666762	N/A	LC666713	Nakao et al. 2022
M. hakubaensis MH2	adult	*Mesocricetus auratus*	*Lethenteron reissneri*	Japan	N/A	KM061389	KM061398	KM061417	Porn 2015
M. katsuradai MK1	adult	*Mesocricetus auratus*	*Tanakia limbata*	Japan	N/A	KM061391	KM061400	KM061418	Pornruseetairatn et al. 2016
M. kinoi NS094	redia	N/A	*Semisulcospira libertina*	Japan, Hokkaido	N/A	LC599533	N/A	LC599824	Nakao et al. 2022
M. kogai Koc4	mtc.	N/A	*Plecoglossus altivelis*	Japan, Shimanto	N/A	LC666749	N/A	LC666573	Nakao et al. 2022
M. kogai NS053	redia	N/A	*Semisulcospira libertina*	Japan, Nagayama-shinkawa	N/A	LC599531	N/A	LC599531	Nakao et al. 2022
M. kogai NS212	redia	N/A	*Semisulcospira libertina*	Japan, Nagayama-shinkawa	N/A	LC599532	N/A	LC599823	Nakao et al. 2022
M. miyatai H008	mtc.	N/A	*Pseudaspius sachalinensis*	Japan, Asahikawa	N/A	LC666744	N/A	LC666468	Nakao et al. 2022
M. miyatai NS027	redia	N/A	*Semisulcospira libertina*	Japan, Nagayama-shinkawa	N/A	LC599529	N/A	LC599811	Nakao et al. 2022
M. miyatai Mm1	adult	*Mesocricetus auratus*	*Plecoglossus altivelis*	Japan, Miyakoda River	HQ832624	HQ832633	HQ832615	N/A	Pornruseetairatn et al. 2016
M. otsurui MO1	adult	*Mesocricetus auratus*	*Rhinogobius flumineus*	Japan	N/A	KM061394	KM061404	KM061423	Pornruseetairatn et al. 2016
M. otsurui MO2	adult	*Mesocricetus auratus*	*Rhinogobius flumineus*	Japan	N/A	KM061395	KM061405	KM061422	Pornruseetairatn et al. 2016
M. otsurui Ky061	mtc.	N/A	*Rhinogobius kurodai*	Japan, Kitakyushu	N/A	LC666757	N/A	LC666630	Nakao et al. 2022
M. otsurui Ky066/Ky064	mtc.	N/A	*Rhinogobius kurodai*	Japan, Kitakyushu	N/A	LC666758	N/A	LC666631	Nakao et al. 2022

M. pusillus 15.I	adult	*Anas platyrhynchos*	*Rhodeus sericeus*	Russia, Bolshaya Ussurka River	N/A	MF407172	MF407172	MF406209	Tatonova et al. 2017
M. romanicus HU24-2	adult	*Mesocricetus auratus*	*Squalius cephalus*	Hungary, Szentendre	PP378514	PP378508	N/A	PP375978	This study
M. romanicus HU24-3	adult	*Mesocricetus auratus*	*Squalius cephalus*	Hungary, Szentendre	N/A	PP378509	N/A	PP375979	This study
M. romanicus HU24-4	adult	*Mesocricetus auratus*	*Squalius cephalus*	Hungary, Szentendre	N/A	PP378510	N/A	PP375980	This study
M. romanicus HU13-1	adult	*Mesocricetus auratus*	*Chondrostoma nasus*	Hungary, Szentendre	N/A	PP378511	N/A	PP375981	This study
M. romanicus HU13-2	adult	*Mesocricetus auratus*	*Chondrostoma nasus*	Hungary, Szentendre	N/A	PP378512	N/A	PP375982	This study
M. romanicus HU13-3	adult	*Mesocricetus auratus*	*Chondrostoma nasus*	Hungary, Szentendre	N/A	PP378513	N/A	PP375983	This study
*M. romanicus*** MM2	mtc.	N/A	*Scaridinius erythrophthalmus*	Hungary, Szentendre	N/A	OQ286071	OQ286093	OQ281688	Cech et al. 2023
*M. romanicus*** ME1	adult	*Gallus gallus*	*Alburnus alburnus*	Hungary, Danube	N/A	OQ286072	OQ286094	OQ281689	Cech et al. 2023
*M. romanicus*** ME3	adult	*Gallus gallus*	*Alburnus alburnus*	Hungary, Danube	N/A	OQ286074	OQ286096	OQ281690	Cech et al. 2023
M. saitoi Ky010/Ky011	mtc.	N/A	*Opsariichthys platypus*	Japan, Kanzaki	N/A	LC666746	N/A	LC666528	Nakao et al. 2022
M. shimazui Ky053	mtc.	N/A	*Acheilognathus rhombeus*	Japan, Kitakyushu	N/A	LC666753	N/A	LC666618	Nakao et al. 2022
M. shimazui OK05	mtc.	N/A	*Acheilognathus rhombeus*	Japan, Okayama	N/A	LC666754	N/A	LC666620	Nakao et al. 2022
M. suifunensis 09.II	adult	*Rattus norvegicus*	*Parajuga subextensa*	Russia, Komissarovka	N/A	KX387459	KX387484	MK736830	Shumenko et al. 2017, Tatonova et al. 2019
M. takahashii H131	mtc.	N/A	*Carassius* sp.	Japan, Asahikawa	N/A	LC666747	N/A	LC666542	Nakao et al. 2022
M. takahashii Mt1	adult	*Mesocricetus auratus*	*Carassius langsdorfii*	Japan, Kiso River	HQ832627	HQ832636	HQ832618	N/A	Pornruseetairatn et al. 2016
M. yokogawai Himi25/Himi27	mtc.	N/A	*Pseudaspius hakonensis*	Japan, Himi	N/A	LC666759	N/A	LC666651	Nakao et al. 2022
M. yokogawai My1	adult	*Mesocricetus auratus*	*Plecoglossus altivelis*	Japan, Sakuma	HQ832630	HQ832639	HQ832621	N/A	Pornruseetairatn et al. 2016
M. yokogawai My2	adult	*Mesocricetus auratus*	*Plecoglossus altivelis*	Japan, Sakuma	HQ832631	HQ832640	HQ832622	N/A	Pornruseetairatn et al. 2016

**Outgroup**									
Amphimerus ovalis	adult	N/A	*Apalone mutica*	U.S.A., Mississippi	AY222121	AY116876	N/A	N/A	Olson et al. 2003
Apophallus muehlingi	adult	N/A	*Larus cachinnans*	Russia, North Caspian region	OK384548	OK358936	N/A	N/A	Sokolov et al. 2021
Clonorchis sinensis	adult	N/A	*Homo sapiens*	Vietnam	JF823988	JF823989	N/A	N/A	Thaenkham et al. 2011
Cryptocotyle lingua	cercaria	N/A	*Littorina littorea*	Germany	AJ287492	AY222228	N/A	N/A	Littlewood & Olson 2000
Euryhelmis costaricensis	mtc.	N/A	*Hynobius lichenatus*	Japan, Minamisoma	AB521797	AB521797	N/A	N/A	Sato et al. 2010
Opisthorchis viverrini	adult	*Mesocricetus auratus*	*Puntius brevis*	Thailand	HM004211	HM004188	N/A	N/A	Thaenkham et al. 2010
Metagonimoides oregonensis	cercaria	N/A	*Pleurocera proxima*	U.S.A., North Carolina	N/A	JQ995473	N/A	N/A	Belden et al. 2012

mtc. – metacercaria; *as *Dexiogonimus ciureanus* in GenBank; ** as *Metagonimus* sp. in GenBank
